# A Three-Decade Analysis of Ischemic Stroke in India: Mortality, Morbidity, and Risk Factors Using the Global Burden of Diseases Study from 1990 to 2019

**DOI:** 10.3390/jcm14217807

**Published:** 2025-11-03

**Authors:** Aditya D. Goyal, Avi A. Gajjar, Najib Muhammad, Albert Q. Wu, Hanish Polavarapu, Oliver Tang, Mohamed M. Salem, Ethan D. Paliwoda, Nithin Gupta, Jagroop Doad, Brian T. Jankowitz, Visish M. Srinivasan, Jan-Karl Burkhardt

**Affiliations:** 1 Department of Neurosurgery, Albany Medical College, Albany, NY 12208, USA; goyala@amc.edu (A.D.G.); paliwode@union.edu (E.D.P.); 2 Department of Neurosurgery, Perelman School of Medicine, University of Pennsylvania, Philadelphia, PA 19104, USA; muhammadn2@upmc.edu (N.M.); albert.wu@pennmedicine.upenn.edu (A.Q.W.); mohamed.salem@cuanschutz.edu (M.M.S.); visish.srinivasan@pennmedicine.upenn.edu (V.M.S.); 3 Department of Neurological Surgery, University of Pittsburgh Medical Center, Pittsburgh, PA 15260, USA; oliveryoungtang@gmail.com; 4 Department of Neurosurgery, SUNY Upstate Medical University, Syracuse, NY 13210, USA; polavarh@upstate.edu; 5 School of Osteopathic Medicine, Campbell University, Lillington, NC 27546, USA; n_gupta0210@email.campbell.edu (N.G.);; 6 Department of Neurosurgery, JFK University Hospital, Edison, NJ 08820, USA; brian.jankowitz@hmhn.org

**Keywords:** ischemic stroke, India, global burden of disease, mortality, morbidity, risk factors

## Abstract

**Background/Objectives**: India has experienced a sharp increase in stroke burden over the last half-century. The diverse geographical conditions and developing health infrastructure warrant an investigation into changes in mortality and morbidity due to ischemic stroke. This research aims to estimate the impact of attributable risk factors, providing comprehensive insights into the temporal trends of ischemic stroke in India. **Methods:** Data regarding ischemic stroke in India were queried from the 2019 Global Burden of Disease (GBD) study. Age-standardized deaths, disability-adjusted life years (DALYs), years lived with disability (YLDs), years of life lost (YLLs), prevalence, and incidence were collected and analyzed. Descriptive statistics and 95% uncertainty intervals (UI) were utilized to ensure reliability. **Results**: In 2019, there were 535,700 incident stroke cases in India (95% CI 453,200–631,800), marking a 118.8% increase from 1990. Females saw a higher incidence rise (131.1%) than males (107.5%). The incidence rate reached 38.5 per 100,000 (95% CI 32.6–45.4), a 34.6% increase, while the age-standardized incidence rate declined by 4.2%. Stroke deaths rose by 148.5%, totaling 271,200 (95% CI 227,800–320,700). Females experienced a higher increase (183.5%) than males (123.0%). Prevalence increased by 130.4%, reaching 6,465,700 cases (95% CI 5,541,000–7,378,000), while age-standardized prevalence rose by 5.7%. Disability-adjusted life years (DALYs) increased by 121.0% to 5,689,300 (95% CI 4,821,100–6,649,500), but the age-standardized DALY rate fell by 23.1%. Years lived with disability (YLD) increased by 135.5% to 907,000 (95% CI 640,300–1,172,900). Years of life lost (YLL) rose by 118.4% to 4,782,300 (95% CI 3,945,500–5,743,700), but the age-standardized YLL rate fell by 26.3%. Metabolic risks, ambient particulate matter pollution, and tobacco smoking were significant risk factors contributing to stroke burden. **Conclusions**: This study highlights contrasting trends in ischemic stroke indicators in India from 1990 to 2019. While prevalence and YLDs have increased, the overall decline in mortality, YLLs, DALYs, and stroke incidence points to improvements in healthcare access and treatment. There is a need for targeted public health interventions to address the growing stroke burden, mainly focusing on preventive measures and addressing sex-based disparities.

## 1. Introduction

Stroke is one of the leading causes of death worldwide, with a disproportionate number of fatalities occurring in underdeveloped regions [1,2]. The Global Burden of Disease (GBD) study is an ongoing observational epidemiological study led by the Institute for Health Metrics and Evaluation that incorporates data on mortality and morbidity across 204 countries, including 369 diseases and injuries and 87 risk factors [3]. This study has been used to assess the global impact of diseases and risk factors over time. In India, the incidence of stroke is on the rise, driven by factors such as insufficient physical activity and a high prevalence of diabetes [1,2]. Low- and middle-income countries, including India, carry a disproportionate share of stroke-related morbidity and mortality due to delayed recognition, inadequate access to acute interventions, and limited rehabilitation infrastructure. The country’s rising life expectancy further exacerbates this increasing trend [4].

Ischemic stroke accounts for the majority of stroke cases in India and globally, representing approximately 70 to 80 percent of all stroke events [5]. Although hemorrhagic stroke carries a higher case-fatality rate, ischemic stroke produces a greater cumulative burden because of its higher incidence and long-term disability impact [5]. It is also more strongly linked to preventable and treatable vascular risk factors, including elevated systolic blood pressure, hyperglycemia, and dyslipidemia, which underscores its suitability as a target for national prevention strategies [2,5]. Despite the introduction of organized stroke services in select regions of India, such as thrombolysis-capable centers and stroke units, access to timely treatment remains uneven nationwide. These disparities highlight the need for detailed, population-level analyses of ischemic stroke trends. Understanding the temporal patterns of ischemic stroke in India can offer essential insights for tailored public health interventions. Previous studies leveraging the GBD dataset have effectively examined stroke trends at various geographic levels [5,6]. In India, lifestyle choices and environmental risk factors contribute significantly to stroke risk, accounting for approximately 90% of cases [5,7]. Given these high-risk indicators, a longitudinal assessment of ischemic stroke in the Indian populace is imperative.

Previous studies using GBD data have examined the global and regional burden of stroke [5,8]. However, few have focused exclusively on ischemic stroke in India with simultaneous reporting of incidence, mortality, and disability indicators along with attributable risk estimates. Lifestyle, metabolic, and environmental factors account for nearly 90 percent of the stroke burden in India [5], which suggests substantial opportunities for prevention if population-level trends can be adequately monitored and addressed. This study utilizes data from the GBD 2019 to analyze the incidence, prevalence, mortality, disability-adjusted life years (DALYs), years of life lost (YLLs), and years lived with disability (YLDs) associated with ischemic stroke in India from 1990 to 2019. Additionally, this research aims to estimate the impact of attributable risk factors, thus providing comprehensive insights into the temporal trends of ischemic stroke in India.

## 2. Materials and Methods

This study utilized data from the Global Burden of Disease Study 2019 (GBD 2019), conducted by the Institute for Health Metrics and Evaluation (IHME), to assess the temporal trends of ischemic stroke in India from 1990 to 2019. The GBD framework systematically compiles global data on disease burden using a unified, comprehensive, and standardized approach across countries and time. The GBD database integrates data from multiple sources, including vital registration systems, household surveys, hospital records, census data, disease registries, and published and unpublished literature.

### 2.1. Case Definition and Metrics

Ischemic stroke was defined according to the World Health Organization (WHO) criteria as a neurological deficit attributed to focal cerebral infarction due to thrombosis, embolism, or systemic hypoperfusion, with imaging or autopsy confirmation where available. Incident cases were limited to first-ever strokes, consistent with GBD classification. We extracted national-level data for both sexes and all age groups in India across six main metrics: incidence, prevalence, mortality, years of life lost (YLLs), years lived with disability (YLDs), and disability-adjusted life years (DALYs) (Table 1).

### 2.2. Computation of Disease Burden Metrics

DALYs were computed as the sum of YLLs and YLDs. YLLs were calculated by multiplying the number of stroke-related deaths by the standard life expectancy at the age of death, based on the GBD reference life table. YLDs were derived by multiplying the prevalence of stroke sequelae by disability weights assigned to each level of functional outcome, as informed by community surveys and expert opinion. Disability weights reflect the severity of health loss and are scaled from 0 (perfect health) to 1 (equivalent to death).

### 2.3. Risk Factor Attribution

Estimates of risk factor contributions were obtained directly from the GBD 2019 results, which use a standardized Comparative Risk Assessment (CRA) framework to attribute portions of disease burden to specific exposures [3]. These risk factors include metabolic (e.g., high systolic blood pressure, high fasting plasma glucose, high BMI, high LDL cholesterol), behavioral (e.g., tobacco use, alcohol consumption, low intake of fruits and vegetables), and environmental exposures (e.g., ambient particulate matter pollution, household air pollution). Population attributable fractions (PAFs) were estimated for each risk factor using exposure distributions, relative risk estimates from meta-analyses, and theoretical minimum risk exposure levels.

### 2.4. Statistical Analysis

Age-standardized rates were calculated using the GBD standard population to enable temporal and sex-specific comparisons. Sex-based comparisons were derived directly from age-standardized rates reported by GBD 2019 [3]. Differences between sexes were interpreted using non-overlapping 95% uncertainty intervals, in accordance with established GBD methodology. All estimates were accompanied by 95% uncertainty intervals (UIs), derived from 1000 draws from the posterior distribution of each model to capture the uncertainty inherent in data inputs and modeling strategies.

All statistical analyses were conducted using Stata 17.0 (StataCorp LLC, College Station, TX, USA). We reported absolute numbers, age-standardized rates per 100,000 population, and percentage changes from 1990 to 2019. No primary data collection was performed, and only anonymized, publicly available data were used; therefore, institutional review board (IRB) approval was not required.

## 3. Results

### 3.1. Incidence

**Figure 1:**
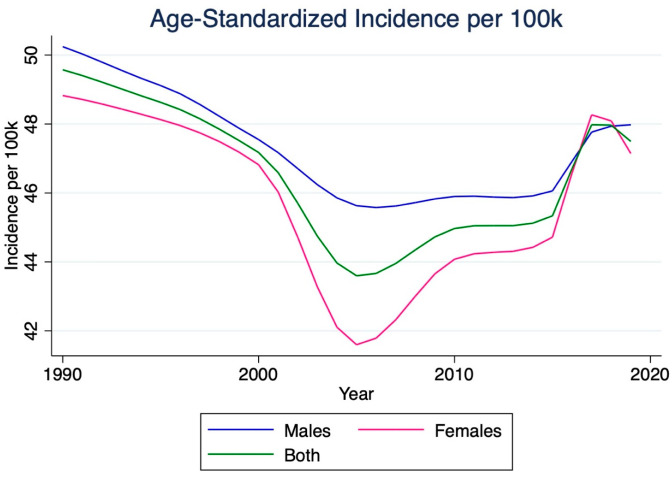
Temporal trends in the age-standardized incidence rate of ischemic stroke in India, 1990–2019. This figure depicts the change in age-standardized incidence of ischemic stroke per 100,000 population over three decades. Although total incident cases increased, the age-standardized rate showed a gradual decline until the mid-2000s, followed by a modest upward trend thereafter. Values represent modeled estimates from the Global Burden of Disease (GBD) 2019 dataset, with shaded areas indicating 95% uncertainty intervals (UIs).

In 2019, there were 535,700 (95% CI 453,200–631,800) incident cases of stroke in India. Stroke incidence increased by 118.8% from 1990 to 2019 (Table 1). Females experienced a more significant increase in incidence (131.1%) compared to males (107.5%). The incidence rate per 100,000 individuals increased by 34.6% from 1990, reaching 38.5 (95% CI 32.6–45.4) per 100,000 in 2019. The increase in incidence rate was more significant for women (40.2%) than for men (29.4%). However, the age-standardized incidence rate per 100,000 individuals decreased by 4.2% from 1990 to 2019, falling to 47.5 (95% CI 40.2–55.7). The decrease was slightly smaller for women (−3.5%) than for men (−4.4%). Despite overall declines in age-standardized stroke incidence rates during this period, there was a converging uptick after 2005 based on sex (Figure 1).

### 3.2. Mortality

**Figure 2:**
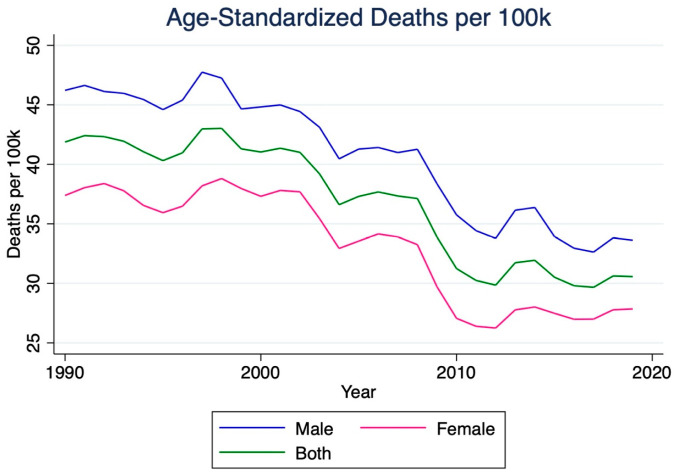
Age-standardized mortality rate for ischemic stroke in India, 1990–2019. This figure illustrates the temporal decline in ischemic stroke mortality per 100,000 population. Although total stroke deaths rose substantially during the study period, age-standardized mortality decreased by approximately 27%, reflecting improved healthcare access and management. Males consistently demonstrated higher mortality rates than females throughout the study period.

The number of stroke deaths in India rose by 148.5% between 1990 and 2019, reaching 271,200 deaths (95% CI 227,800–320,700). Females experienced a more significant increase in fatalities (183.5%) compared to males (123.0%). The age-standardized mortality rate per 100,000 individuals decreased by 27.0% from 1990 to 2019. Overall, stroke mortality rates experienced a net decline between 1990 and 2019. However, males consistently exhibited higher mortality rates than females (Figure 2).

### 3.3. Prevalence

**Figure 3:**
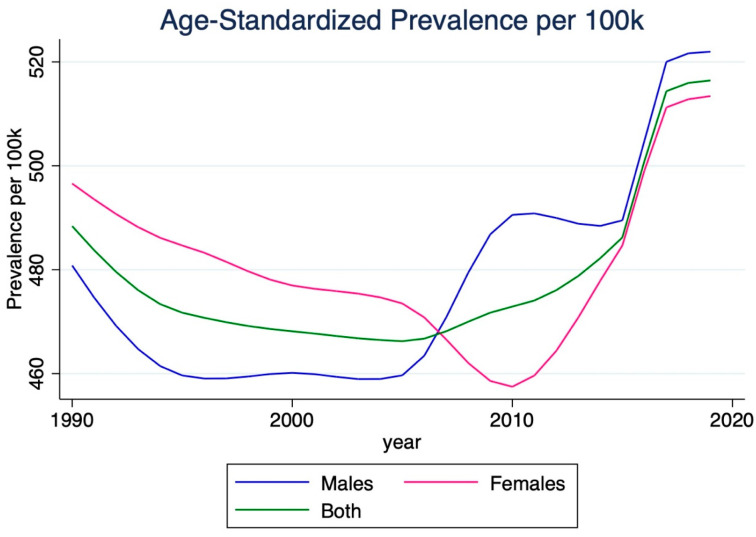
Age-standardized prevalence of ischemic stroke in India, 1990–2019. The plot shows the steady increase in prevalence of ischemic stroke over the 30-year period. While the crude prevalence rose markedly, age-standardized prevalence increased modestly, suggesting improvements in survival and chronic disease management. Convergence of male and female prevalence rates after 2005 indicates narrowing sex differences in disease burden.

The number of strokes in India significantly increased by 130.4% from 1990 to 2019, reaching 6,465,700 (95% CI 5,541,000–7,378,000). The corresponding stroke prevalence rate rose 41.7% over this period, reaching 464.9 per 100,000 individuals (95% CI 398.4–530.5). The age-standardized prevalence rate increased by 5.7%, reaching 516.4 per 100,000 individuals (95% CI 443.5–590.0). Men experienced a more significant increase in this rate (8.6%) than women (3.4%). Before 2005, women typically experienced a higher stroke prevalence rate per 100k than men. However, after 2005, males began to experience a rapid rise in prevalence rates, leading to converging rates for both sexes (Figure 3).

### 3.4. Disability-Adjusted Life Years (DALYs)

**Figure 4:**
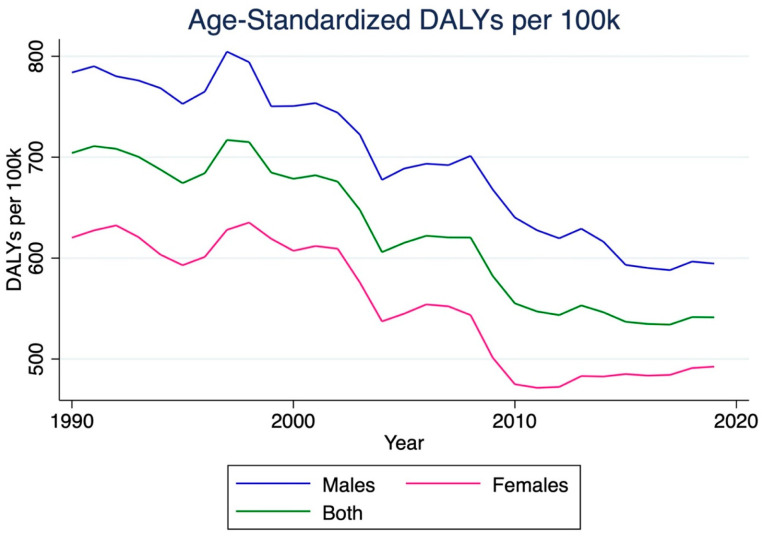
Trends in age-standardized disability-adjusted life years (DALYs) due to ischemic stroke in India, 1990–2019. This figure displays the change in DALY rates per 100,000 population, summarizing both fatal and non-fatal health loss attributable to ischemic stroke. Despite an absolute increase in DALYs, the age-standardized DALY rate declined over time, suggesting partial success of stroke prevention and management initiatives.

The number of stroke disability-adjusted life years (DALYs) in India increased by 121.0% between 1990 and 2019, reaching a total of 5,689,300 (95% CI 4,821,100–6,649,500) (Table 2). Females experienced a more significant increase in stroke DALYs (145.7%) compared to males (102.8%). The stroke DALY rate per 100,000 individuals increased by 36.0%, reaching 409.1 (95% CI 346.7–478.1). Females experienced a more significant increase in this rate (48.8%) than males (26.6%). However, the age-standardized DALYs rate per 100,000 individuals decreased by 23.1%, falling to 541.4 (95% CI 461.6–633.2). Males experienced a slightly more significant decrease (−24.1%) than females (−20.6%). Overall, males consistently exhibited higher stroke DALY rates than females (Figure 4).

### 3.5. Years Lived with Disability (YLDs)

**Figure 5:**
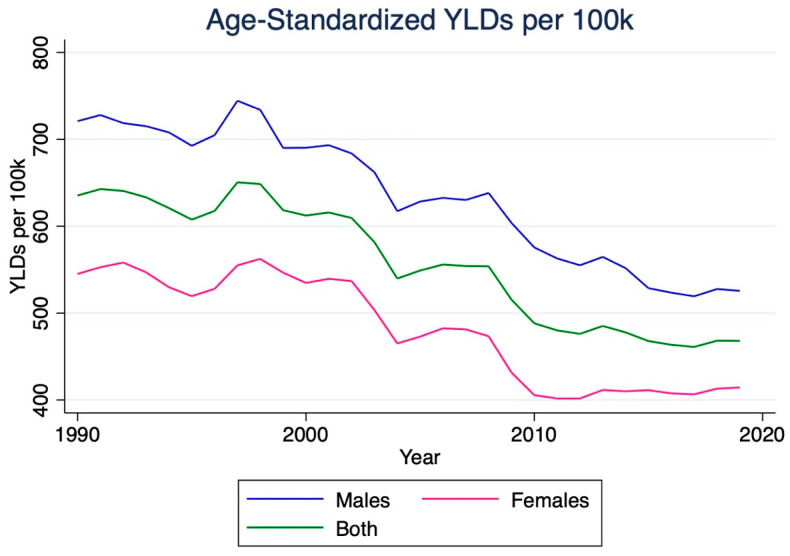
Temporal trends in age-standardized years lived with disability (YLDs) due to ischemic stroke in India, 1990–2019. The figure highlights an upward trajectory in YLDs, representing the growing long-term morbidity associated with ischemic stroke. The gradual increase in age-standardized YLD rates indicates that although survival has improved, stroke survivors face a higher burden of residual disability.

The number of years living with disability (YLD) due to stroke in India increased by 135.5% between 1990 and 2019, reaching 907,000 (95% CI 640,300–1,172,900). The stroke YLD rate per 100,000 individuals rose by 44.9%, reaching 65.2 (95% CI 46.0–84.3). Meanwhile, the age-standardized stroke YLD rate per 100,000 individuals increased by 6.7%, reaching 73.4 (95% CI 51.5–94.9). Men experienced a more significant increase in this rate (9.7%) than women (4.1%). Furthermore, overall stroke YLD rates were consistently higher for males than for females (Figure 5).

### 3.6. Years of Life Lost (YLLs)

**Figure 6:**
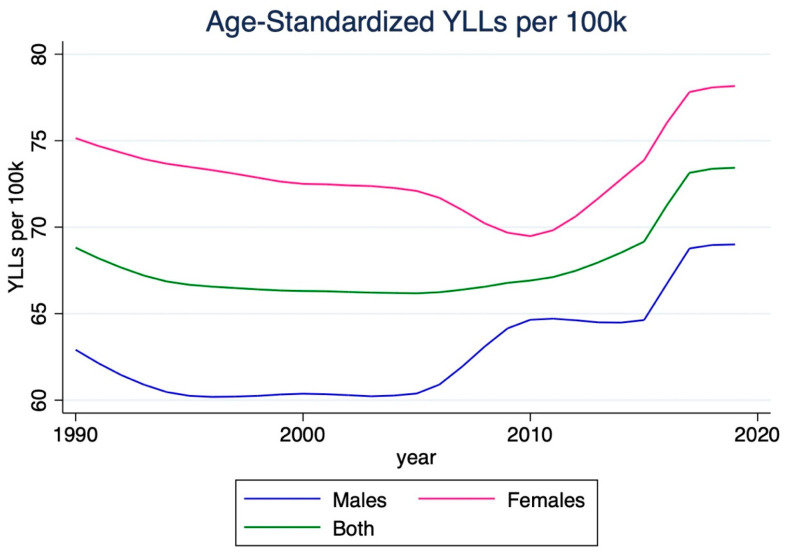
Temporal trends in age-standardized years of life lost (YLLs) due to ischemic stroke in India, 1990–2019. This figure illustrates a substantial absolute increase in YLLs, but a decline in the age-standardized rate, suggesting reductions in premature mortality in relation to population aging. Females exhibited a larger proportional increase in total YLLs, consistent with longer life expectancy and demographic expansion.

The number of years of life lost (YLL) due to stroke increased significantly between 1990 and 2019, with a 118.4% increase, totaling 4,782,300 (95% CI 3,945,500–5,743,700). Females experienced a higher increase (147.0%) than males (98.9%). The stroke YLL rate per 100,000 individuals also increased by 34.4%. Females experienced a more significant increase (49.6%) than males (24.2%). Despite this, the age-standardized stroke YLL rate per 100,000 individuals decreased by 26.3%, reaching 467.9 (95% CI 391.6–559.2). Figure 6 shows that, despite decreases in age-standardized rates, females remain at greater risk of YLL from stroke compared to males.

### 3.7. Risk Factors

In India, the total DALY per 100,000 individuals for all risk factors associated with ischemic stroke patients was 463.8 (95% CI 389.9–547.1) (Table 2). The DALY rate was higher in males (512.8, 95% CI 396.8–658.3) compared to females (418.1, 95% CI 336.1–503.0). The highest specific risk factor for both genders was metabolic risk, with 413.2 (95% CI 307.8–538.8) for males and 347.1 (95% CI 267.6–429.8) for females. The second highest risk factor was ambient particulate matter pollution, with 142.8 (95% CI 99.3–193.9) for males and 103.7 (95% CI 75.5–134.3) for females. Tobacco smoking was also a significant risk factor, contributing 95.5 (95% CI 71.5–126.1) for males and 16.9 (95% CI 12.1–22.5) for females. Secondhand smoke was additionally a risk factor, with 14.4 (95% CI 8.9–21.1) for males and 17.1 (23.4 to 11.7) for females.

Dietary risks were also significant, with a diet high in red meat having a DALY rate of 5 (95% CI 1.6 to 9.4) for males and 3.8 (95% CI 1.3 to 6.9) for females. A diet high in sodium had a DALY rate of 37.3 (95% CI 3.5 to 101.5) for males and 20.7 (95% CI 1.2 to 68.3) for females. A diet low in fruits had a DALY rate of 44.3 (95% CI 13.2 to 80.1) for males and 36.7 (95% CI 12.4 to 62.5) for females. A diet low in vegetables had a DALY rate of 17 (95% CI 2.6 to 32.4) for males and 14.8 (95% CI 2.6 to 28.3) for females.

Alcohol use was also a risk factor, with a DALY rate of 16.3 (95% CI 3.4 to 30.7) for males and −0.7 (95% CI −2.1 to 0.8) for females. Physiological factors were also significant risk factors, with high fasting plasma glucose having a DALY rate of 184 (95% CI 91.7 to 342) for males and 132.2 (95% CI 65.9 to 249.2) for females. High LDL cholesterol had a DALY rate of 98.9 (95% CI 51.4 to 179.8) for males and 91.5 (95% CI 48.7 to 165.4) for females. High systolic blood pressure had a DALY rate of 284.3 (95% CI 203.7 to 386) for males and 246.3 (95% CI 181.8 to 319.9) for females. High body-mass index had a DALY rate of 62.4 (95% CI 31.2 to 105.7) for males and 64.6 (95% CI 35.8 to 99.1) for females. Kidney dysfunction had a DALY rate of 57.7 (95% CI 40.1 to 80.4) for males and 48.2 (95% CI 32.6 to 65.4) for females.

**Table 2:** Attributable ischemic stroke burden by major risk factors in India, 2019. The table reports age-standardized disability-adjusted life years (DALYs) per 100,000 population attributable to specific risk factors for ischemic stroke, categorized by sex. Risk factors are grouped into environmental, behavioral, and metabolic domains, following the GBD Comparative Risk Assessment framework. Population-attributable fractions were estimated based on exposure levels, relative risks, and theoretical minimum risk exposure distributions.

	Male	Female	Total
**All Risk Factors**	512.8 (658.3 to 396.8)	418.1 (503 to 336.1)	463.8 (547.1 to 389.9)
Ambient particulate matter pollution	142.8 (193.9 to 99.3)	103.7 (134.3 to 75.5)	122.6 (154.3 to 92.8)
Household air pollution from solid fuels	74.6 (115.3 to 41.3)	74.7 (106.7 to 49.8)	74.7 (105.4 to 49.2)
High temperature	13.4 (26.1 to 1)	10.7 (19.2 to 0.6)	12 (21.8 to 0.8)
Low temperature	11.3 (25.9 to −2.1)	8.4 (19 to −1.9)	9.8 (21 to −1.9)
Lead exposure	50.2 (74.5 to 31)	36 (51.8 to 22.1)	42.8 (60.6 to 27.4)
**Tobacco Smoking**			
Smoking	95.5 (126.1 to 71.5)	16.9 (22.5 to 12.1)	55 (70.5 to 42.5)
Secondhand smoke	14.4 (21.1 to 8.9)	17.1 (23.4 to 11.7)	15.8 (21.1 to 10.7)
**Dietary Risks**			
Diet high in red meat	5 (9.4 to 1.6)	3.8 (6.9 to 1.3)	4.4 (7.8 to 1.5)
Diet high in sodium	37.3 (101.5 to 3.5)	20.7 (68.3 to 1.2)	28.7 (81.1 to 2.3)
Diet low in fruits	44.3 (80.1 to 13.2)	36.7 (62.5 to 12.4)	40.4 (70.5 to 13)
Diet low in vegetables	17 (32.4 to 2.6)	14.8 (28.3 to 2.6)	15.8 (30 to 2.6)
Diet low in whole grains	26.4 (43.5 to 6.8)	20.1 (31.9 to 4.6)	23.2 (35.9 to 5.7)
Alcohol use	16.3 (30.7 to 3.4)	−0.7 (0.8 to −2.1)	7.5 (14.6 to 1)
**Physiological Factors**			
High fasting plasma glucose	184 (342 to 91.7)	132.2 (249.2 to 65.9)	157.2 (293.2 to 81.4)
High LDL cholesterol	98.9 (179.8 to 51.4)	91.5 (165.4 to 48.7)	95.2 (167.8 to 50.6)
High systolic blood pressure	284.3 (386 to 203.7)	246.3 (319.9 to 181.8)	265 (336.8 to 202.5)
High body mass index	62.4 (105.7 to 31.2)	64.6 (99.1 to 35.8)	63.6 (98.2 to 35.3)
Kidney dysfunction	57.7 (80.4 to 40.1)	48.2 (65.4 to 32.6)	52.9 (70.3 to 38.2)
**Cluster of Risk Factors**			
Air pollution	217.4 (282 to 164.2)	178.4 (217.2 to 139.9)	197.3 (235.3 to 162.2)
Non-optimal temperature	24.5 (42.5 to 9.3)	18.9 (31.3 to 7)	21.6 (35.9 to 8.6)
Tobacco	107.3 (140.3 to 80.1)	33.4 (43.2 to 24.9)	69.2 (86.6 to 54.1)
Dietary risks	131.9 (203.2 to 74.3)	99.6 (148.4 to 59.9)	115.2 (170.7 to 68.8)
Behavioral risks	233.4 (319.1 to 166.3)	142.5 (194 to 96.2)	186.5 (249 to 135.4)
Metabolic risks	413.2 (538.8 to 307.8)	347.1 (429.8 to 267.6)	379.2 (459.2 to 304.9)

## 4. Discussion

This study aimed to examine and delineate trends in the stroke burden faced by the population in India over the past thirty years. Data collected from the GBD 2019 and analyses of six key metrics herein revealed that age-standardized rates of stroke prevalence and YLDs have risen despite an observable overall decline in age-standardized rates of stroke incidence, mortality, DALYs, and YLLs. India has primarily mirrored high-income countries with overall decreases in age-standardized incidence rates, mortality, and DALYs [9,10,11,12]. However, the trends revealed in this study present alarming disparities, highlighting urgent areas for intervention and policy formulation [13]. These findings suggest that, despite improvements in healthcare access and treatment effectiveness, the goal of reducing the stroke burden still faces significant challenges.

**Figure 7:**
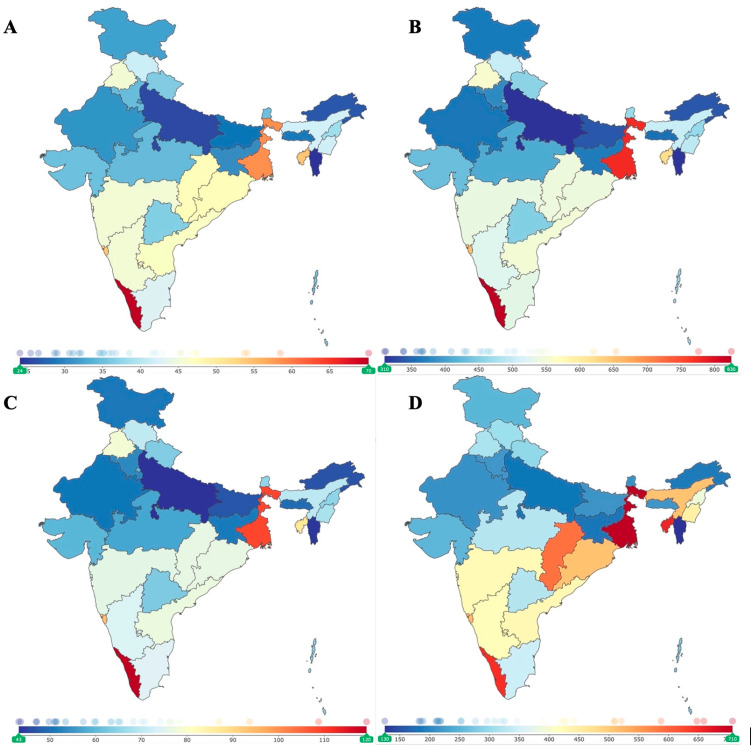
Regional variation in ischemic stroke burden across India, 2019. Panels (**A**–**D**) display subnational differences in age-standardized (**A**) incidence, (**B**) prevalence, (**C**) years lived with disability (YLDs), and (**D**) years of life lost (YLLs) due to ischemic stroke. Geographic heterogeneity reflects underlying disparities in healthcare access, environmental exposures, and population risk factor profiles.

While there has been an overall decrease in stroke incidence in India from 1990 to 2019, the incidence has increased between 2005 and 2019. The rise in incidence during this sub-period, coupled with the increasing prevalence of stroke, is a concerning trend that affects different regions of India in varying ways (Figure 7). This escalating stroke incidence concomitates India’s rapid socio-economic changes, including increased urbanization and lifestyle modifications [14,15,16,17]. Urbanization has been linked with higher stress levels, decreased physical activity, and dietary changes, which collectively exacerbate the primary risk factors for stroke [18]. Moreover, the disparity in healthcare infrastructure between urban and rural areas, as well as between different states, may also contribute to the varied stroke incidence and outcomes observed across the country [19,20].

The gender disparities highlighted in our study suggest deeper systemic issues. Existing literature shows that women tend to have a higher lifetime risk of experiencing a stroke and poorer outcomes after a stroke compared to men [21]. Women’s health, particularly in less developed regions, is often deprioritized, potentially leading to even higher risks and poorer outcomes after stroke events [21]. This situation is compounded by social stigmas that marginalize women’s health issues and lower health literacy, which limits women’s understanding of health risks and available medical options [22,23]. Furthermore, women often face limited autonomy in healthcare decisions, often influenced by cultural norms that restrict their independence in seeking and consenting to medical care [24,25]. Such barriers are not merely logistical but are deeply embedded in the socio-cultural fabric of communities, necessitating a multifaceted approach to intervention. To effectively reduce stroke incidence among women, it is crucial to enhance access to health education tailored to the specific needs of women [26,27]. Interventions to reduce stroke incidence in women must address these systemic and socio-cultural barriers by enhancing women’s access to health education, improving healthcare affordability, and encouraging gender-sensitive and culture-sensitive health policymaking [26].

Addressing the increasing stroke burden requires a dual approach: enhancing clinical care and reinforcing public health prevention strategies. Although India has initiatives in place, such as the National Programme for Prevention and Control of Cancer, Diabetes, Cardiovascular Diseases, and Stroke (NPCDCS), its effectiveness remains questionable [28]. A study by Venugopal et al. examining the NPCDCS in the context of the World Health Organization’s Innovative Care for Chronic Conditions (WHO ICCC) framework revealed significant deficiencies at the individual, organizational, and policy levels [29]. Clinically, there is a pressing need to establish more comprehensive stroke centers equipped with advanced diagnostic and treatment technologies [30]. Increasing the presence of these centers in proximity to rural areas, as well as enhancing accessibility in urban areas, may lead to more evenly distributed healthcare services, thereby bridging the gap between urban and rural areas [31]. However, despite its potential to alleviate the stroke burden, this measure may face challenges from limited resources and inadequate infrastructure in many parts of India [31].

More feasible and impactful public health initiatives include widespread education on stroke risk factors and symptoms, community-based screening programs for hypertension and diabetes, and interventions to promote physical activity and healthy eating [14,32,33]. Legislative measures to control tobacco, manage air quality, and ensure safer occupational environments are equally crucial [32]. Furthermore, despite the inadequacies of the NPCDCS, investing in the proper training and deployment of community health workers (CHWs), particularly in rural regions, may be beneficial. Examining 510 CHWs in a rural South Indian district demonstrated acceptable knowledge and positive practices being implemented in stroke awareness [34]. With appropriate training and strategic use, CHWs have the potential to play a critical role at the frontline in recognizing and responding to stroke symptoms, especially in underserved areas lacking primary care physicians (PCPs) and neurologists [35]. These community-based efforts underscore the pressing need for a comprehensive and multifaceted approach that not only improves access to specialized care but also strengthens the overall healthcare system to combat the increasing burden of stroke across India.

The trends observed in India reflect a broader global epidemiological transition, wherein stroke burden is increasingly shifting toward low- and middle-income countries despite advances in prevention and care [36,37]. This pattern underscores the need for India to strengthen national surveillance and integrate stroke prevention into primary health care, consistent with the World Stroke Organization’s call for context-specific, data-driven policy frameworks [37]. Future progress depends on improving the continuity of care from acute management to secondary prevention, particularly through scalable strategies such as tele-neurology, community-based rehabilitation, and hypertension control programs [37]. Importantly, a sustainable reduction in stroke burden will require investment in real-time data systems and consistent participation in the Global Burden of Disease (GBD) framework to monitor evolving risk patterns and assess the impact of interventions. Aligning national policy with these global priorities represents a pivotal step toward mitigating the growing health and economic consequences of stroke in India.

### Limitations

Our research presents multiple limitations that should be acknowledged. First, accurately identifying stroke-related deaths is challenging, particularly when distinguishing them from fatalities caused by comorbidities. The study also lacks the TOAST classification, which would enable a more nuanced clinical examination of ischemic stroke severity; future research should incorporate this for a thorough analysis. Regional estimates may be biased due to heterogeneous data sources from different areas. Furthermore, the uncertainty intervals may not fully represent variability, especially in less-represented, remote regions; increasing the robustness of health surveys in these locations is essential for precision. Specific analysis of disease burden by age group was deemed beyond the scope of the present analysis. Future work focused on the age-related disease burden of AIS must provide an age-specific subgroup analysis of GBD data.

Additionally, this study did not consider prevailing stroke prevention and treatment methods, an element that needs to be included in subsequent analyses. Economic disparity alone does not successfully explain divergent stroke burdens across regions. Lastly, the complex interactions among different stroke risk factors need to be more thoroughly understood and further examined.

## 5. Conclusions

In conclusion, our analysis of GBD data from 1990 to 2019 revealed that while age-standardized stroke incidence, mortality, DALYs, and YLLs have decreased, age-standardized stroke prevalence and YLDs have increased. This trend underscores the evolving nature of the stroke burden in India. Additionally, significant sex-based disparities in stroke outcomes highlight the need for targeted interventions. Further research is essential to comprehend the underlying causes of these disparities and develop effective public health strategies to reduce the stroke burden in India. Strengthening healthcare infrastructure, enhancing preventive measures, and addressing socio-cultural barriers are critical steps toward achieving this goal.

## Figures and Tables

**Table 1:** Summary of ischemic stroke indicators in India by sex, 1990–2019. This table presents the number and age-standardized rates (per 100,000 population) of incidence, mortality, prevalence, disability-adjusted life years (DALYs), years lived with disability (YLDs), and years of life lost (YLLs) for ischemic stroke in 2019, along with percent changes since 1990. Data are stratified by sex. Percent changes were calculated using modeled estimates from the GBD 2019 database. Negative values indicate reductions in age-standardized rates over time. Abbreviations: DALYs = disability-adjusted life years; YLDs = years lived with disability; YLLs = years of life lost; UI = uncertainty interval.

	2019	Percent Change, 1990–2019	2019	Percent Change, 1990–2019
	Incidence (95% UI), thousands		Age-standardized Incidence per 100k (95% UI)	
Male	264.1 (222.1 to 311.9)	107.5	48 (40.6 to 56.4)	−4.4
Female	271.6 (229.4 to 320.7)	131.1	47.1 (39.8 to 55.3)	−3.5
Total	535.7 (453.2 to 631.8)	118.8	47.5 (40.2 to 55.7)	−4.2
	Deaths (95% UI), thousands		Age-standardized mortality per 100k (95% UI)	
Male	141 (107.5 to 180.3)	123.0	33.6 (26.3 to 42.5)	−27.3
Female	130.2 (100.7 to 160.4)	183.5	27.8 (21.6 to 34)	−25.7
Total	271.2 (227.8 to 320.7)	148.5	30.6 (25.7 to 35.8)	−27.0
	Prevalence (95% UI), thousands		Age-standardized Prevalence per 100k (95% UI)	
Male	3215.1 (2745.7 to 3677.6)	125.4	522 (448.8 to 599)	8.6
Female	3250.6 (2790.2 to 3702.2)	135.6	513.4 (444.6 to 584)	3.4
Total	6465.7 (5541 to 7378)	130.4	516.4 (443.5 to 590)	5.7
	DALYs (95% UI), thousands		Age-standardized DALYs per 100k (95% UI)	
Male	3009.1 (2320.9 to 3853.5)	102.8	594.6 (466.9 to 753.6)	−24.1
Female	2680.2 (2154.8 to 3217.5)	145.7	492.5 (395.3 to 587)	−20.6
Total	5689.3 (4821.1 to 6649.5)	121.0	541.4 (461.6 to 633.2)	−23.1
	YLDs (95% UI), thousands		Age-standardized YLDs per 100k (95% UI)	
Male	416.8 (288.9 to 542.2)	130.4	69 (48.1 to 89.5)	9.7
Female	490.3 (345.2 to 632.1)	140.0	78.2 (55.6 to 100.7)	4.1
Total	907 (640.3 to 1172.9)	135.5	73.4 (51.5 to 94.9)	6.7
	YLLs (95% UI), thousands		Age-standardized YLLs per 100k (95% UI)	
Male	2592.3 (1906.7 to 3412.8)	98.9	525.6 (395.9 to 679.8)	−27.1
Female	2189.9 (1688 to 2710.5)	147.0	414.3 (320.2 to 509.7)	−24.0
Total	4782.3 (3945.5 to 5743.7)	118.4	467.9 (391.6 to 559.2)	−26.3

## Data Availability

Data are available upon reasonable request to the corresponding author.

## Abbreviations

The following abbreviations are used in this manuscript:

GBD	Global Burden of Disease
DALYs	Disability-Adjusted Life Years
YLDs (YLD)	Years Lived with Disability
YLLs (YLL)	Years of Life Lost
UI	Uncertainty Interval
CI	Confidence Interval
WHO	World Health Organization
CODEm	Cause of Death Ensemble Model
DisMod-MR 2.1	Disease Modeling–Meta Regression, version 2.1
BMI	Body Mass Index
LDL	Low-Density Lipoprotein
PAF(s)	Population Attributable Fraction(s)
IRB	Institutional Review Board
NPCDCS	National Programme for Prevention and Control of Cancer, Diabetes, Cardiovascular Diseases, and Stroke
WHO ICCC	World Health Organization Innovative Care for Chronic Conditions
CHWs	Community Health Workers
PCPs	Primary Care Physicians
TOAST	Trial of ORG 10172 in Acute Stroke Treatment
